# Biomarcadores en la enfermedad de Alzheimer

**DOI:** 10.1515/almed-2020-0109

**Published:** 2021-01-27

**Authors:** Manuel H. Janeiro, Carlos G. Ardanaz, Noemí Sola-Sevilla, Jinya Dong, María Cortés-Erice, Maite Solas, Elena Puerta, María J. Ramírez

**Affiliations:** Departamento de Farmacología y Toxicología, Facultad de Farmacia y Nutrición, Universidad de Navarra, Pamplona, España; IDISNA, Instituto de Investigaciones Sanitarias de Navarra, Pamplona, España

**Keywords:** enfermedad de Alzheimer, sangre, líquido cefalorraquídeo, amiloide b, deterioro cognitivo leve, déficit cognitivo, neuroimagen

## Abstract

**Objetivos:**

La enfermedad de Alzheimer (EA) es una enfermedad neurodegenerativa. La EA es la principal causa de demencia en el mundo, siendo el envejecimiento el principal factor de riesgo. Los criterios diagnósticos para la enfermedad de Alzheimer suelen basarse en datos clínicos. No obstante, es necesario establecer una definición biológica de la enfermedad de Alzheimer basada en biomarcadores que reflejen la neuropatología subyacente.

**Contenido:**

El objetivo de esta revisión es presentar los resultados obtenidos en la medición de biomarcadores nuevos y ya conocidos en los fluidos biológicos o en neuroimágenes.

**Resumen:**

Actualmente se emplean tres biomarcadores para el diagnóstico de la enfermedad de Alzheimer_Aβ42, t-Tau y p-Tau. El uso diagnóstico de biomarcadores en el líquido cefalorraquídeo (LCR) presenta algunas limitaciones debido a que la obtención invasiva mediante punción lumbar puede provocar efectos secundarios. La práctica más común en los centros clínicos es la medición en plasma o suero, ya que es mínimamente invasiva y, en consecuencia, se puede obtener y procesar con mayor facilidad. Las dos principales proteínas implicadas en el proceso patológico, Aβ y Tau, se pueden visualizar empleando técnicas de neuroimagen como la PET.

**Perspectivas:**

Dado que está ampliamente aceptado que la enfermedad de Alzheimer comienza décadas antes de que se diagnostiquen los primeros síntomas clínicos, la detección de alteraciones biológicas previa a la aparición de la sintomatología clínica permitiría su diagnóstico precoz o incluso abriría la puerta a nuevas opciones terapéuticas.

## Introducción

La enfermedad de Alzheimer (EA) es una enfermedad neurodegenerativa y la principal causa de demencia en el mundo, siendo el envejecimiento el principal factor de riesgo. Se estima que actualmente hay en todo el mundo 46,8 millones de personas con demencia y se prevé que esta cifra irá aumentando con el envejecimiento de la población mundial, alcanzando los 74,7 millones de personas. Actualmente, solo cuatro fármacos has sido aprobados por la FDA para tratar la enfermedad: tres inhibidores de la colinesterasa (donepezil, rivastigmina y galantamina) y un modulador del receptor de N-metil-D-aspartato (NMDA) no competitivo (memantina). Por desgracia, ninguno de estos fármacos retrasa o detiene el avance de la enfermedad. Por lo tanto, la enfermedad de Alzheimer se ha convertido en uno de los principales retos sanitarios del siglo.

**Figura 1:**
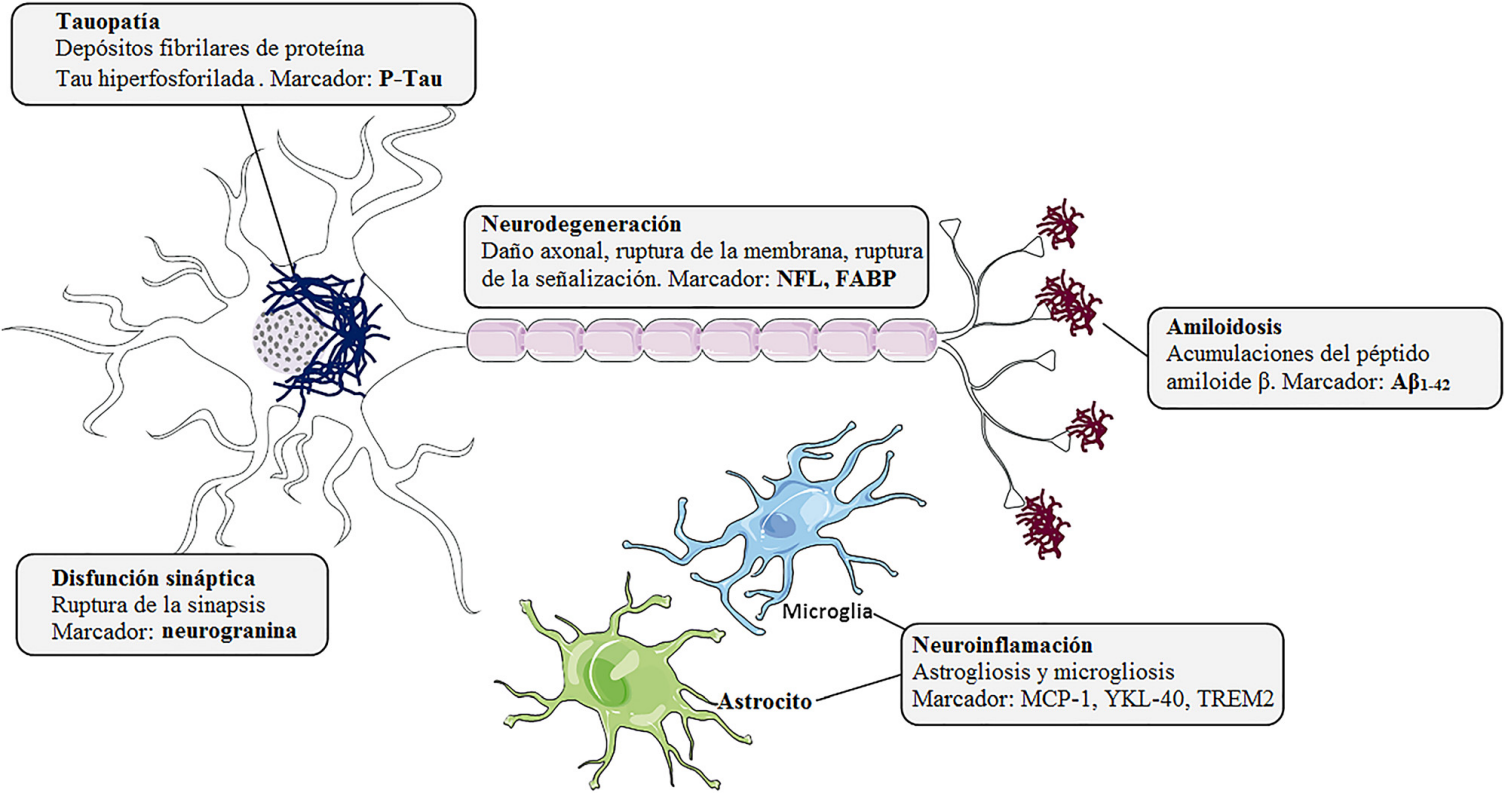
Marcadores biológicos de alteraciones histopatológicas en la enfermedad de Alzheimer. Placas seniles, compuestas por acumulaciones del péptido amiloide β (Aβ), ovillos neurofibrilares (NFT) formados por depósitos fibrilares de la proteína Tau hiperfosforilada (p-Tau), neuroinflamación, disfunción sináptica y neurodegeneración.

Clínicamente, la EA se caracteriza por el deterioro de la memoria y las funciones cognitivas. La mayoría de los pacientes presentan síntomas neuropsiquiátricos llamados “síntomas conductuales y psicológicos de demencia”, como la depresión, la excesiva actividad, la psicosis o las conductas agresivas. Las características histológicas de la EA son las placas seniles, compuestas por depósitos del péptido beta amiloide (Aβ) y por ovillos neurofibrilares (NFT, por sus siglas en inglés), que son depósitos fibrilares de la proteína Tau hiperfosforilada (p-Tau). Otros eventos patológicos que parecen jugar un papel esencial en la enfermedad son la disfunción sináptica, la inflamación o la alteración vascular (Figura 1).

Los criterios diagnósticos de la EA suelen basarse en información clínica. No obstante, es necesario establecer una definición biológica de la EA basada en biomarcadores que reflejen la neuropatología subyacente. En un síndrome tan complejo como la EA, los biomarcadores serían de utilidad para el diagnóstico temprano, la estadificación de la enfermedad, el establecimiento de un pronóstico y la evaluación de la respuesta al tratamiento. Además, los biomarcadores ayudarían a comprender los mecanismos de la enfermedad y a desarrollar nuevas estrategias de tratamiento.

**Figura 2:**
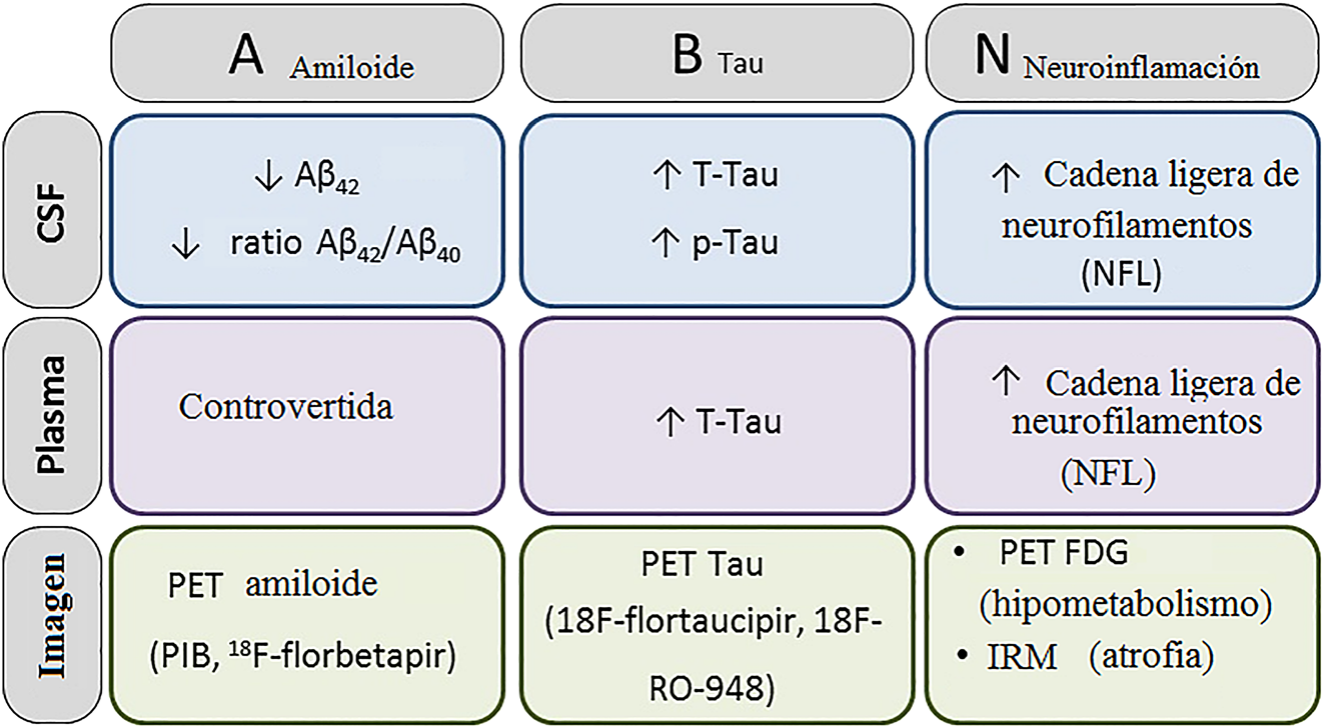
La clasificación de neurodegeneración basada en la proteína tau y el amiloide (AT(N)) define como biomarcadores “A” a la tomografía por emisión de positrones amiloide (PET), y a los niveles de Aβ42 y Aβ 42/40 en el líquido cefalorraquídeo (LCR). Los biomarcadores “T” son la proteína tau en PET y p-Tau en LCR, mientras que los biomarcadores “N” son la resonancia magnética estructural, el PET con fluorodesoxiglucosa (FDG), la tau total en LCR (t-Tau) y la proteína de cadena ligera de neurofilamentos (NFL).

Según las últimas guías de práctica clínica del Instituto Nacional de Envejecimiento y la Asociación de Alzheimer (NIA-AA) [1], el término “enfermedad de Alzheimer” se emplea ante la presencia de biomarcadores que muestran la existencia de placas de Aβ y NFT. Actualmente (Figura 2), la clasificación de neurodegeneración basada en la proteína tau y el amiloide (AT(N)) define como biomarcadores “A” a la tomografía por emisión de positrones amiloide (PET), y a los niveles de Aβ42 y Aβ 42/40 en el líquido cefalorraquídeo (LCR). Los biomarcadores “T” son la proteína tau en PET y p-Tau en LCR, mientras que los biomarcadores “N” son la resonancia magnética estructural, el PET con fluorodesoxiglucosa (FDG), la tau total en LCR (t-Tau) y la proteína de cadena ligera de neurofilamentos (NFL) [2]. El propósito de esta revisión es describir los principales valores obtenidos en la determinación de biomarcadores nuevos y conocidos en fluidos biológicos o en los estudios de neuroimagen.

## Biomarcadores en fluidos: el líquido cefalorraquídeo

El LCR refleja los procesos metabólicos del cerebro, ya que este se encuentra en contacto directo con el cerebro. De este modo, el LCR resulta muy útil en el diagnóstico de la EA. Los biomarcadores en el LCR tienen más interés que los biomarcadores en plasma, ya que muestran una mayor correlación con los resultados de la PET realizada con el compuesto B de Pittsburgh marcado con 11C, mostrando además mayor valor predictivo [3]. Así mismo, aumenta la precisión del diagnóstico, especialmente en pacientes con enfermedad en fase prodrómica o con presentaciones atípicas [4]. Actualmente se emplean tres biomarcadores del LCR para el diagnóstico de la EA: Aβ42, t-Tau y p-Tau.

### Pacientes con Alzheimer frente a los controles sanos y pacientes con otras patologías del sistema nervioso central (SNC)

Los péptidos Aβ como Aβ42, Aβ40, Aβ37, Aβ38, sAPPα, y sAPPβ fueron los primeros biomarcardores moleculares identificados [5]. Entre ellos, Aβ42 está especialmente relacionado con los síntomas fisiológicos y patológicos, ya que los pacientes con Alzheimer muestran menores niveles de Aβ42 en el LCR que los sujetos sanos debido a los depósitos de amiloide cortical. Por el contrario, los niveles de Aβ42 en plasma no muestran esta asociación [3]. Se ha demostrado una asociación entre niveles bajos de Aβ40 y la enfermedad de Alzheimer. De todos modos, su pequeño efecto de tamaño limita su uso en el diagnóstico de la enfermedad. Aunque se han observado diferencias significativas entre pacientes con Alzheimer y los controles, su efecto es mínimo. Además, Aβ37 y Aβ38 podrían ayudar a distinguir la EA de otros tipos de demencia similares [6], aunque no son realmente útiles por sí solos para el diagnóstico clínico, ya que no se han hallado diferencias significativas entre los pacientes y los controles en diversos estudios [3].

sAPPα y sAPPβ son productos de la escisión de la proteína precursora del amiloide amiloidea (APP, por sus siglas en inglés). Los niveles de sAPPα parecen ser inferiores en el síndrome parkinsoniano atípico o el Parkinson, lo que sugiere una alteración del metabolismo de la APP [7]. Sin embargo, sAPPα y sAPPβ no resultan muy útiles por sí solos en el diagnóstico clínico, ya que no se han observado diferencias significativas entre pacientes con Alzheimer y controles. No obstante, si se analiza el sAPPα junto con otros biomarcadores en el LCR, se puede mejorar la precisión diagnóstica [3]. Posteriormente, t-Tau y p-Tau (Thr 181) fueron aceptados como biomarcadores. Estos marcadores están relacionados con problemas de memoria y están sobreexpresados en el LCR de los pacientes con Alzheimer. Mecanísticamente, t-Tau podría elevarse debido a la pérdida de neuronas corticales, por lo que la elevación de p-Tau indicaría la formación de ovillos corticales. Cabe mencionar que la presencia de p-Tau es específica de la EA, por lo que resultaría útil a la hora de distinguir esta enfermedad de otros tipos de demencia [3].

Cuando estos tres marcadores se determinan conjuntamente (Aβ42, t-Tau and p-Tau), se logra una mayor especifidad y sensibilidad, que cuando se determina uno solo de ellos [5]. Aunque en menor medida, en el déficit cognitivo leve también se encuentran alteraciones en estos marcadores, lo que no ocurre en el deterioro cognitivo leve estable [5]. Estos biomarcadores pueden ayudar a predecir la progresión del déficit cognitivo leve a Alzheimer. El principal problema a la hora de emplear estos biomarcadores es que, debido a la variabilidad entre laboratorios, no se han consensuado unos valores de referencia para definir los niveles bajos de Aβ42 y los niveles elevados de t-Tau [3].

Además de los biomarcadores ya conocidos, en estudios recientes se han identificado nuevos marcadores [5]. Existe un creciente interés en los marcadores asociados a la neurodegeneración, como la cadena ligera de neurofilamentos (NFL). La NFL, junto con el medio o la copia de cadena pesada, forma los haces de neurofilamentos que determinan el diámetro de los axones y la velocidad de conducción [8]. Se ha observado que la NFL está sobreexpresada en los axones mielinizados. Así mismo, cuando se produce degradación o degeneración axonal, se libera NFL al LCR y al torrente sanguíneo. Niveles elevados de NFL pueden ser indicativos de que se está produciendo daño axonal y desmielinización [9]. En varios estudios, se ha mostrado una relación significativa entre los niveles de NFL y un gran efecto de tamaño indicativo de que existe una destrucción axonal importante en la EA [5]. A mayores niveles de NFL, mayor gravedad de la enfermedad, aunque no están correlacionados con la duración de la enfermedad. Además, la NFL puede contribuir a mejorar la precisión diagnóstica, ya que puede ayudar a diferenciar la demencia con cuerpos de Lewy (DCL) y la EA de la demencia de Parkinson, ya que en la DCL se produce un incremento de NFL [7]. La edad también está asociada a niveles elevados de NFL [10].

YKL-40, un marcador relacionado con la activación glial y astrocítica, así como otras proteínas como la carnosinasa I, la cromogranina A y la molécula de adhesión de células neuronales (NCAM) podría ser útil para el diagnóstico precoz [3], aunque tiene un tamaño de efecto moderado [5]. Por otro lado, no se ha demostrado que la proteína quimioatrayente de monocitos-1 (MCP-1) o la proporción de albúmina sean marcadores diagnósticos [5].

Algunos estudios han revelado la presencia de niveles ligeramente superiores de α-sinucleína en pacientes con Alzheimer [11]. Esta hipótesis contrasta con el hecho de que los pacientes con Párkinson presentan menores niveles de α-sinucleína en el LCR, comparados con los controles. Cabe destacar que, en una elevada proporción de los estudios que mostraron un aumento de los niveles de α-sinucleína en el LCR en pacientes con Alzheimer, el diagnóstico se estableció únicamente conforme a criterios clínicos, por lo que resulta imposible dilucidar si los pacientes presentaban enfermedad de cuerpos de Lewy concomitante.

Finalmente, se han obtenido resultados prometedores con otros biomarcadores del LCR como la neurogranina o el TREM-2. En los últimos cinco años, se han publicado varios estudios que demuestran que los pacientes con Alzhéimer presentan niveles elevados de la proteína postsináptica específica neurogranina (expresada principalmente en las regiones afectadas por la EA como la corteza, el hipocampo y la amígdala), siendo esta sobreexpresión específica de la EA, ya que no ha sido detectada en otras enfermedades neurodegenerativas. Este incremento podría reflejar degeneración de las sinapsis [12]. Por otra parte, algunos estudios recientes han revelado que el TREM2, que es un receptor inmune innato que se expresa en la superficie de la microglía y se procesa proteolíticamente y se libera como fragmento soluble (sTREM2), está aumentado en la EA. Además, se ha observado que existe una relación entre niveles elevados de sTREM2 en el LCR y mayores niveles de tau total y p-Tau (Thr-181) [13]. Aunque solo se han publicado unos pocos estudios, la literatura sugiere que estos niveles podrían estar aumentados en etapas tempranas y alcanzar valores máximos antes de que se desarrolle la demencia.

En resumen (Tabla 1), los biomarcadores que han mostrado mayor consistencia hasta la fecha y deberían ser empleados en la práctica clínica, así como en los estudios de investigación, son t-Tau, p-Tau, y Aβ42 en el LCR, que han mostrado una precisión de entre el 85 y el 90%. Además de estos marcadores conocidos y basándonos en la evidencia científica, la NFL se postula como un nuevo biomarcador. Del mismo modo, la neurogranina en el LCR ha arrojado resultados prometedores, ya que posee una gran especifidad y un gran tamaño de efecto, aunque es necesario realizar más estudios para verificar su precisión y utilidad.

**Tabla 1:** Biomarcadores de la enfermedad de Alzheimer.

Biomarcadores	Descripción	LCR	Plasma	Utilidad diagnóstica
Aβ42	Marcador del metabolismo del APP	↓ niveles en pacientes con Alzheimer. Gran tamaño del efecto	No hay diferencias	Recomendado para el diagnóstico mediante el análisis de LCR
Aβ40	Marcador del metabolismo del APP	↓ niveles en pacientes con Alzheimer. Pequeño tamaño del efecto	No hay diferencias	No muy útil por sí solo.
Aβ38	Marcador del metabolismo del APP	No hay diferencias entre grupos		No muy útil por sí solo.Puede ayudar a distinguir el Alzheimer de otras formas similares de demencia.
sAPPα	Producto de escisión de la APP	No hay diferencias entre grupos		No muy útil por sí solo.
sAPPβ	Producto de escisión de la APP	No hay diferencias entre grupos		No muy útil por sí solo.
t-Tau y p-Tau (Thr 181)	Marcadores relacionados con problemas de memoria	↑ niveles en pacientes con Alzheimer. Gran tamaño del efecto	↑ niveles de t-Tau en pacientes mediante el análisis de Alzheimer. Tamaño del efecto grande	p-Tau es característica del Alzheimer. Recomendado para el diagnóstico mediante el análisis de LCR
NFL	Marcador relacionado con la neurodegeneración	↑ niveles en pacientes con Alzheimer. Gran tamaño del efecto		Recomendado para el diagnóstico mediante el análisis de LCR
NSE	Marcador relacionado con la neurodegeneración	↑ niveles en pacientes con Alzheimer. Tamaño del efecto moderado	No hay diferencias	Podría ser utilizado en el diagnóstico mediante el análisis de LCR
VLP-1	Marcador relacionado con la neurodegeneración	↑ niveles en pacientes con Alzheimer. Tamaño del efecto moderado		Podría ser utilizado en el diagnóstico mediante el análisis de LCR
HFABP	Marcador relacionado con la neurodegeneración	↑ niveles en pacientes con Alzheimer. Tamaño del efecto moderado	No hay diferencias	Podría ser utilizado en el diagnóstico mediante el análisis de LCR
Ratio de albúmina	Marcador de la función de la barrera hematoencefalica	↑ niveles en pacientes con Alzheimer. Pequeño tamaño del efecto		No muy útil por sí solo.
YKL-40	Marcador de la activación de las células gliales	↑ niveles en pacientes con Alzheimer. Tamaño del efecto moderado	↑ niveles en pacientes con Alzheimer. Gran tamaño del efecto, aunque no significativo	Podría ser útil en el diagnóstico mediante el análisis de LCR
MCP-1	Marcador de la activación de las células gliales	↑ niveles en pacientes con Alzheimer. Pequeño tamaño del efecto	No hay diferencias	No muy útil por sí solo.
GFAP	Marcador de la activación de las células gliales	No hay diferencias entre grupos		No muy útil por sí solo.
Neurogranina	Marcador de la degeneración sináptica	↑ niveles en pacientes con Alzheimer. Gran tamaño del efecto		Específico del Alzheimer. Muy prometedor, pero pocos estudios publicados.
sTREM2	Marcador relacionado con la neurodegeneración	↑ niveles en pacientes con Alzheimer. Tamaño del efecto moderado		Podría ser útil en el diagnóstico mediante el análisis de LCR, pero hay pocos estudios publicados.
α-sinucleína	Proteína neuronal presináptica	↑ niveles en pacientes con Alzheimer. Tamaño de efecto mínimo.		No muy útil por sí solo. La mayoría de los estudios se ha realizado en pacientes con alta probabilidad de Alzheimer

↑:aumento ↓:reducción. Datos extraidos de Janelidze y col, 2016 [14], Majbour, 2017 [11], Olsson y col., 2016 [5], Suárez-Calvet, 2016 [13] y Wellingtonet al., 2016 [12].

### Pacientes con Alzheimer frente a los pacientes con deterioro cognitivo leve

La utilidad de los biomarcadores anteriormente mencionados varía a la hora de comparar a los pacientes con Alzheimer de aquellos con deterioro cognitivo leve (DCL) (Tabla 2). Únicamente t-Tau and p-Tau, que se mantienen elevados en los pacientes con Alzheimer, conservan un gran tamaño de efecto. Por otro lado, los niveles de Aβ42 siguen siendo bajos en los pacientes con Alzheimer, aunque con un menor tamaño de efecto. Sin embargo, aunque los niveles de Aβ40 en el LCR se redujeron levemente frente a los controles, no se observaron diferencias con respecto a los pacientes con DCL. Además, Aβ38, que no varió en los pacientes con Alzheimer con respecto a los controles, aumentó mínimamente con respecto a los pacientes con DCL [5].

**Tabla 2:** Biomarcadores en pacientes con DCL- Alzheimer y DCL estable.

Biomarcadores	LCR	Plasma	Utilidad diagnóstica
Aβ42	↓ niveles en pacientes con Alzheimer. Menor tamaño del efecto que entre los pacientes con Alzheimer y sujetos sanos.	No hay diferencias.	Uso recomendado.
Aβ40	No hay diferencias.	↑ niveles en pacientes con Alzheimer. Tamaño del efecto mínimo.	No muy útil.
Aβ38	↑ niveles en pacientes con Alzheimer. Tamaño de efecto mínimo.		Solo dos estudios publicados. Pequeño tamaño del efecto.
sAPPα	No hay diferencias		No muy útil.
sAPPβ	No hay diferencias		No muy útil.
t-Tau y p-Tau (Thr 181)	↑ niveles de t-Tau y p-Tau en los pacientes con Alzheimer. Tamaño del efecto grande.		Uso recomendado.
Neurogranina	↑ niveles en pacientes con Alzheimer. Tamaño del efecto moderado.		Nuevo y prometedor biomarcador relativo. Pocos estudios publicados.
YKL-40	↑ niveles en pacientes con Alzheimer. Tamaño del efecto moderado.		Nuevo y prometedor biomarcador relativo. Pocos estudios publicados.

↑:aumento ↓:reducción. Datos extraidos de Janelidze y col., 2016 [14] y Olsson y col, 2016 [5]. DCL, deterioro cognitivo leve.

Finalmente, los niveles de neurogranina y YKL-40 también fueron superiores, con un tamaño de efecto moderado en los pacientes con Alzheimer, comparados con aquellos con DCL [14], aunque hasta la fecha únicamente se han publicado cuatro estudios al respecto.

En conclusión, los mejores biomarcadores a la hora de distinguir a los pacientes con Alzheimer de aquellos con DCL son p-Tau y t-Tau, aunque la neurogranina y el YKL-40 también podrían resultar útiles.

## Biomarcadores en fluidos: sangre

El uso diagnóstico de biomarcadores en el líquido cefalorraquídeo (LCR) presenta algunas limitaciones, debido a los efectos secundarios que puede provocar la obtención invasiva de LCR mediante punción lumbar. En este contexto, se han dirigido los esfuerzos a descubrir biomarcadores fiables en sangre. La práctica más común en los centros clínicos es la determinación en plasma o suero, ya que es mínimamente invasiva y, en consecuencia, se puede obtener y procesar con mayor facilidad [15]. Además, se puede realizar un seguimiento y cribado de los pacientes a largo plazo. No obstante, por diferentes razones, la identificación de biomarcadores de la EA en sangre ha resultado ser más compleja que en el LCR. En primer lugar, los cambios son muy pequeños y heterogéneos, ya que los datos de plasma y suero reflejan un amplio espectro de cambios, no necesariamente relacionados con la EA. En segundo lugar, solo una fracción de las proteínas cerebrales llega al torrente sanguíneo, debiendo ser estas determinadas en una matriz con altas concentraciones de proteínas plasmáticas, como la albúmina y la IgG, existiendo un importante riesgo de interferencia entre los métodos analíticos [16]. Además de diluirse, las proteínas cerebrales liberadas al torrente sanguíneo pueden ser degradadas por las proteasas, metabolizadas en el hígado o eliminadas por los riñones, lo que introduce una variable ajena a los cambios cerebrales que es difícil de controlar [17]. Por otro lado, a excepción de algunos biomarcadores sanguíneos como el Aβ plasmático [18] y el tau plasmático [19], aún existen pocos estudios sobre la relación entre las proteínas en el LCR y sus análogos sanguíneos en la misma cohorte. Así, la precisión con la que los cambios moleculares periféricos reflejan las dinámicas del SNC aún no se ha determinado a gran escala [20].

### Niveles de Aβ

Aunque en numerosos estudios se ha observado una buena concordancia con la determinación del número de placas en PET amiloide, así como una reducción significativa de Aβ42 en el LCR de pacientes con Alzheimer, no se han obtenido resultados coherentes con respecto al uso de Aβ42 plasmático como biomarcador. En esta línea, diferentes estudios revelan que los niveles plasmáticos de Aβ42 y Aβ40 pueden estar elevados, reducidos o inalterados al comparar a pacientes con Alzheimer con controles sanos [21], [22]. Así mismo, aunque en estudios longitudinales previos se ha mostrado que los niveles aumentados de Aβ42 en plasma son un factor de riesgo de desarrollar Alzheimer [23], en otros se ha asociado una proporción baja de Aβ42/Aβ40 con un mayor riesgo inminente de desarrollar deterioro cognitivo leve y Alzheimer [24]. De este modo, existe cierto consenso en que este factor no tiene suficiente sensibilidad ni especifidad para el diagnóstico temprano [5].

Además, no parece existir correlación entre el LCR y los niveles plasmáticos de Aβ [25], lo que sustenta la hipótesis de que los niveles plasmáticos de Aβ se corresponden con la producción de Aβ en la sangre periférica desde otros tejidos, más que con la patología cerebral de la EA. Así mismo, los niveles de Aβ fluctúan con el tiempo y varían de un individuo a otro [15]. Al unirse a otras proteínas y quedar atrapada [26], la expresión de Aβ en plasma puede verse influida por el efecto de los fármacos [27] y, lo que es más importante, las plaquetas contienen altos niveles de Aβ, lo que afecta a los niveles plasmáticos de Aβ [28].

Además, esta falta de relación podría ser el resultado de las limitaciones metodológicas de los métodos ELISA u otros inmunoensayos frecuentemente utilizados [29]. Algunos estudios han revelado que, debido a su hidrofobia, los péptidos de Aβ interactúan con numerosas proteínas de la matriz de plasma, como la albúmina, la macroglobulina-β2 o las lipoproteínas, entre otras [26]. Esto podría enmascarar al epítote, impidiendo el reconocimiento de hasta el 50% de estos péptidos amiloides en los inmunoensayos [30]. Por lo tanto, este efecto de la matriz podría afectar a la fiabilidad de las determinaciones del péptido Aβ en un sujeto. En este contexto, el equipo de Zetterberg desarrolló en 2011 un nuevo método basado en la técnica de Simoa, en la que se emplean matrices capaces de detectar moléculas individuales, para determinar el Aβ42 plasmático [31]. Esta técnica, basada en la inmunocaptura del biomarcador proteico en perlas magnéticas, seguido por la introducción de anticuerpos de captura marcados con enzimas, permite cuantificar con exactitud los niveles de Aβ42 con una alta sensibilidad, reduciendo las interferencias de la matriz. El estudio sueco de cohortes BioFINDER probó esta técnica y reveló una proporción Aβ42 plasmático/40 inferior tanto en sujetos con DCL como con Alzheimer, frente a los sujetos control [32]. Además, los mismos autores desarrollaron un método de monitorización de reacción seleccionada mediante espectrometría de masas por inmunoprecipitación para la cuantificación de Aβ42 y Aβ40 plasmático. En un pequeño estudio clínico piloto en el que se empleó esta misma técnica, solo los sujetos con Alzheimer mostraron tendencia a presentar niveles inferiores de Aβ42 plasmático, así como una menor proporción de Aβ42/40 [33]. Cabe mencionar que, empleando el mismo método de IP-MS, los casos con PET amiloide positiva mostraron concentraciones significativamente inferiores de Aβ42 así como una proporción de Aβ42/40 significativamente menor, frente a los casos con PET negativa [34].

Otros estudios basados en la espectrometría de masas sugieren que la proporción de un fragmento de APP concreto (APP669-711) con respecto a Aβ42 o Aβ40/Ab42 en plasma identifica a los individuos Aβ-positivos con una alta sensibilidad y especifidad [35]. Concretamente, la proporciones de APP669-711/Aβ42 y Aβ40/Aβ42 fueron mayores en los individuos Aβ-positivos que en los Aβ-negativos [35]. Estos prometedores resultados abren la puerta a nuevos estudios con cohortes clínicas más amplias destinados a evaluar el Aβ plasmático como herramienta de detección de la amiloidosis cerebral y la EA.

### Proteína tau

Teniendo en cuenta todos los biomarcadores séricos y plasmáticos, el t-Tau es el único que discrimina a los pacientes con EA de los controles, tal como se ha demostrado en la mayoría de los estudios publicados [5], mostrando un menor incremento del tau plasmático en los pacientes con EA aunque con un solapamiento excesivo con los controles, lo que impide que sea una herramienta diagnóstica [19]. Curiosamente, los datos longitudinales han mostrado correlaciones significativas entre los niveles plasmáticos de tau y un posterior deterioro cognitivo, así como mayor atrofia determinada mediante RM, e hipermetabolismo, determinado mediante FDG-PET durante el seguimiento [19]. Además, se ha descubierto que la proteína tau está presente en el LCR en forma de fragmentos truncados [36], por lo que es posible que el desarrollo de técnicas analíticas basadas en anticuerpos específicos de estos fragmentos de tau mejore su rendimiento como biomarcador. Por otro lado, la determinación de t-Tau o p-Tau en preparaciones de exosomas enriquecidas con neuronas podría mejorar el rendimiento de tau como biomarcador en sangre [37], aunque son necesarios más estudios para validar este hallazgo.

### Proteína NFL

Actualmente, la proteína NFL es el biomarcador en sangre más replicado en la EA. El primer método Simoa para la cuantificación de la proteína axonal NFL en muestras de sangre se publicó en 2016 [38]. Así, numerosos estudios han mostrado mayores concentraciones de NFL en pacientes con EA comparados con sujetos sanos de la misma edad [39], mientras que otros estudios sugieren que se podría utilizar la cuantificación de NFL en sangre como biomarcador de neurodegeneración en fases preclínicas de EA.

Aunque el cambio en el grupo de DCL fue menos pronunciado, estos pacientes fueron los que presentaron mayores concentraciones de NFL plasmático, con estudios PET amioloide positivos, lo que predijo un deterioro cognitivo más rápido, mayor índice de atrofia cerebral en el futuro (determinada mediante RM) e hipermetabolismo, determinado mediante FDG-PET [39]. Además, en los estudios sobre EA hereditario, NFL parece estar alterado alrededor de una década antes del inicio de los síntomas [40], existiendo una correlación entre los niveles de NFL y el año estimado de inicio de los síntomas, el deterioro cognitivo y la fase de la enfermedad determinada mediante RM [41].

No obstante, es importante destacar que el NFL no es una característica específica de la EA. Esta proteína está elevada en muchas otras patologías neurodegenerativas, como la demencia frontotemporal, la parálisis supranuclear progresiva, el síndrome corticobasal, las afecciones inflamatorias o la lesión cerebral traumática aguda [42]. Por tanto, aunque NFL carece de especifidad diagnóstica, con la determinación semiautomática de NFL en sangre se pueden tomar múltiples muestras de sangre para realizar un seguimiento de la evolución de la enfermedad y monitorizar la respuesta al tratamiento. La NFL plasmática también se podría emplear en el futuro en un test de detección simple, no invasivo y barato, para la evaluación clínica inicial de los pacientes con problemas cognitivos, principalmente para descartar la enfermedad neurodegenerativa.

## Biomarcadores de imagen: PET-PiB, PET-FDG, IRM

Las tecnologías de imagen nos han permitido comprender mejor las complejas interrelaciones entre los mecanismos patofisiológicos de la EA. Las dos principales proteínas implicadas en el proceso patológico, Aβ y tau, se pueden visualizar con técnicas como la PET. Además, el impacto de la neurodegeneración a nivel local se puede caracterizar minuciosamente. Así, los defectos en el metabolismo de la glucosa cerebral, la atrofia en regiones cerebrales y las alteraciones de la red cerebral se pueden evaluar mediante PET, resonancia magnética (IRM) estructural y MRI funcional, respectivamente. De este modo, un estudio detallado de las imágenes de la dinámica espaciotemporal de la patofisiología de la EA ofrecería más información sobre la biología y la evolución de la enfermedad en pacientes concretos.

### PET amiloide

Los radiofármacos como PIB o 18F-florbetapir poseen una elevada especifidad y sensibilidad para la unión a placas amiloides. Así, el PET con amiloides permite visualizar las formas agregadas de Aβ (aunque es incapaz de detectar las formas solubles oligoméricas más patogénicas). Existe un amplio consenso sobre el notable incremento en la acumulación de Aβ en el cerebro afectado por la EA. El mapeo de Aβ cerebral ha revelado que algunas áreas amplias del corteza de asociación medial y lateral tienen mayor predisposición a acumular Aβ, incluso en los individuos sin demencia [43]. Cabe destacar que en la primera etapa de desarrollo de la EA, parece producirse una acumulación de Aβ en la corteza parietal medial. En los pacientes con enfermedad de Alzheimer autosómica dominante (DIAD), parece que la acumulación de Aβ se produce en el cuerpo estriado y el lóbulo temporal medial hasta 20 años antes de la aparición de los primeros síntomas [44]. En el caso de la EA en el envejecimiento, la acumulación de Aβ comienza en la corteza parietal medial y frontal [43]. La acumulación de Aβ se produce en la corteza de asociación, aunque no está correlacionada a los síntomas [45]. El PET amiloide es la única técnica de neuroimagen para la que se están publicando valores de referencia más o menos sólidos: un estudio muy exhaustivo en el que se comparan diversos métodos para establecer valores de referencia señala un valor de amiloide de 19 en PET en la escala centiloide como un buen valor límite para la EA [46].

### PET-tau

Dada la relativa novedad de los ligandos de PET-tau, no existen muchos estudios sobre la concentración de tau. No obstante, tras la primera generación de trazadores de tau (p.ej. 18F-flortaucipir), cuya mayor limitación era su unión con otras dianas, se desarrolló una segunda generación (18F-RO-948) de trazadores, con los que se están realizando fructíferos estudios sobre la dinámica espaciotemporal de la acumulación de tau. Aunque la cronología y localización física de la patología de tau son inciertas, esta podría desarrollarse inicialmente en el lóbulo temporal medial. Así, en pacientes con Alzheimer, se han identificado mayores niveles de retención de trazadores de tau en diferentes regiones cerebrales, entre las que se encuentran el lóbulo temporal medial y la región temporal lateral inferior, el surco parietal lateral y el giro cingulado posterior [47], [48]. El uso de trazadores de tau de segunda generación ha permitido confirmar que los pacientes con Alzheimer muestran una señal de trazadores más intensa en las áreas temporales mediales, en el giro cingulado posterior, los lóbulos parietal lateral y occipital, y la corteza prefrontal [49]. Sin embargo, apenas se han realizado estudios experimentales o de validación clínica de los trazadores de tau [50], y aún se desconoce la cantidad de tau hiperfosforilada que hay presentes en el cerebro, que se encuentra por debajo de los límites de detección. Por diferentes razones, aún no se ha establecido un valor de referencia fiable para el diagnóstico de la EA con PET tau [46].

### PET FDG

El metabolismo de la glucosa en el cerebro se puede mapear con un PET empleando el análogo de la glucosa FDG. De este modo, la FDG permite caracterizar la actividad metabólica, cuyos déficits se suelen interpretar como pérdida de la función neuronal y/o sináptica, aunque también pueden reflejar alteraciones en la función de las células gliales, que han adquirido una importancia creciente en los estudios sobre la neuropatología de la EA. La localización espacial del hipometabolismo difiere entre los diversos síndromes que componen la EA lo que brinda la oportunidad de identificar el fenotipo: comienza en el lóbulo temporal medial años antes de la aparición de los primeros síntomas de la enfermedad, para extenderse a la corteza parietal lateral en el DIAD [51]. Está fuertemente correlacionada con la acumulación de tau en la EA de inicio temprano [52] y predomina en el lóbulo temporal medial y en el lóbulo parietal medial en la EA de inicio tardío [53]. Por el contrario, el hipometabolismo no sigue un patrón específico en el envejecimiento normal, a pesar de mostrar predominancia frontal. Finalmente, con el avance de la enfermedad, se produce un patrón topográfico de hipometabolismo del cerebro muy característico en la corteza temporal parietal y el giro cingulado posterior [54]. Sin embargo, aún no se ha dilucidado el grado y localización del hipometabolismo necesarios para establecer un valor límite fiable en los pacientes con Alzheimer.

### IRM estructural

Dependiendo de la señal originada en el interior del cuerpo, la IRM estructural permite determinar de forma precisa y no invasiva el volumen de los tejidos del cerebro, lo que permite localizar características patofisiológicas como la atrofia cerebral regional, la pérdida de materia gris o el daño en la sustancia blanca. Entre los principales hallazgos realizados con las técnicas de medición volumétrica del cerebro, se ha identificado la atrofia de la materia gris en las estructuras del lóbulo temporal medial en sujetos con Alzheimer, aunque también en aquellos con DCL. Al parecer, el biomarcador más fiable de la progresión de DCL a Alzheimer es la atrofia del lóbulo temporal medial izquierdo [55]. Otros parámetros que predicen la progresión de DCL a Alzheimer son la reducción del volúmen de materia gris en el hipocampo y el parahipocampo y la presencia más difusa de pérdida cortical en la EA [56]. Además, las técnicas de difusión, que aprovechan que la difusión microscópica del agua es anisotrópica en el cerebro humano, lo que permite identificar tractos de sustancia blanca, han detectado el deterioro de algunos tractos mayores en la EA. Durante las etapas transicionales del envejecimiento normal al Alzheimer se produce una interrupción del giro cingulado (que conecta las estructuras del lóbulo temporal medial con el resto del cerebro). También se han encontrado lesiones en el uncinate fasciculus, implicado en los procesos emocionales y de la memoria [57]. No obstante, aún se desconoce el número de células cerebrales o de tractos de sustancia blanca que se deben perder para que se pueda detectar o definir la atrofia en la EA, y aún no se dispone de suficientes datos para poder establecer un valor límite fiable de adelgazamiento cortical [46].

### IRM funcional

La técnica de medir las señales de IRM que reflejan la perfusión de los tejidos, es decir la IRM funcional (fMRI), se basa en el fundamento de que las diferentes regiones cerebrales que muestran fluctuaciones sincrónicas de las señales pertenecen a la misma red cerebral. Una interrupción de dichas señales se interpreta como una alteración en la conectividad de la red, lo que permite analizar los cambios en la conectividad cerebral. Existen evidencias de que una pérdida en la conectividad funcional que afecte a regiones críticas de la red neuronal por defecto (RND), como la corteza cingulada posterior, preceden a la pérdida de materia gris en la EA [58]. Dado que otros tipos de demencia implican alteraciones de diferentes redes [59], la interrupción de las señales de conectividad de la RND, determinada mediante fMRI, podría ser un biomarcador indicativo de la EA. Por último, pero no menos importante, la desconexión en el hipocampo y en la corteza prefrontal medial también parecen ser características de la EA [60]. Como algunas de las otras técnicas de imagen descritas, la falta de evidencia ha impedido poder establecer un valor límite para las alteraciones de señales en fMRI que permita diagnosticar o identificar a los pacientes con Alzheimer.

## Perspectivas futuras: nuevos biomarcadores y fluidos

Se están realizando diversos estudios para desarrollar técnicas no invasivas para el análisis de muestras biológicas de fácil acceso como la saliva [61]. Hasta la fecha, los resultados obtenidos no han sido concluyentes, o incluso son contradictorios a la hora de medir Aβ42 [62], [63], [64] o p-Tau [64], [65] en este fluido [66]. Cabe señalar que la variación circadiana podría influir considerablemente en la composición de la saliva. Además, la salud bucal o la medicación podrían afectar a la detección de biomarcadores. De este modo, es necesario establecer un protocolo de trabajo estándar para obtener resultados reproducibles [67].

En los últimos años, el miRNA en sangre ha surgido como un prometedor biomarcador de la EA. Los miRNA son moléculas cortas no codificantes que funcionan como factores epigenéticos que regulan la expresión génica de forma post transcripcional uniéndose a secuencias complementarias en los miRNA diana. El miRNA suele estar encapsulado en los exosomas, microvesículas o cuerpos apopóticos, estructuras que pueden transportar otras sustancias. Los exosomas que contienen miRNA pueden atravesar la barrera hematoencefálica y mediar la comunicación entre la sangre, el cerebro y el LCR [68]. El miRNA desempeña una función fisiológica, pero también patológica, ya que algunos de ellos están alterados en la EA. Los exosomas más ampliamente estudiados en la EA tienen puntos de unión en miRNA que codifican proteínas con un papel fundamental en la enfermedad: APP, tau, BACE, PSEN2, MAPK …etc. En condiciones fisiológicas, los exosomas pueden transportar proteínas acumuladas (Aβ y tau) a los lisosomas o al plasma extracelular para su degradación. Sin embargo, en condiciones patológicas, esta eliminación se ve interrumpida. Sin embargo, aunque no se hayan obtenido resultados concluyentes, no podemos dejar de mencionar una revisión reciente que revela que has-miR-146a, has-miR-125b y hsa-miR135a podrían estar expresadas diferencialmente en la sangre y en el LCR de pacientes con Alzheimer, frente a los sujetos sanos, con otras enfermedades neurológicas o incluso con DCL [68].

## Conclusiones

Aunque los criterios tradicionalmente empleados para el diagnóstico de la EA se basan en evidencias clínicas, es necesario establecer otros criterios con el fin de poder identificar la enfermedad en sus etapas más tempranas. Actualmente, está ampliamente aceptado que la EA comienza décadas antes de la aparición de los primeros síntomas clínicos. La posibilidad de detectar alteraciones biológicas con anterioridad a la aparición de los síntomas clínicos permitiría establecer un diagnóstico temprano o incluso modificar las opciones de tratamiento.
